# A Dimer of the Toll-Like Receptor 4 Cytoplasmic Domain Provides a Specific Scaffold for the Recruitment of Signalling Adaptor Proteins

**DOI:** 10.1371/journal.pone.0000788

**Published:** 2007-08-29

**Authors:** Ricardo Núñez Miguel, Joyce Wong, Julian F. Westoll, Heather J. Brooks, Luke A.J. O'Neill, Nicholas J. Gay, Clare E. Bryant, Tom P. Monie

**Affiliations:** 1 Department of Biochemistry, University of Cambridge, Cambridge, United Kingdom; 2 Department of Veterinary Medicine, University of Cambridge, Cambridge, United Kingdom; 3 School of Biochemistry and Immunology, Trinity College, Dublin, Ireland; University of Helsinki, Finland

## Abstract

The Toll-like receptor 4 (TLR4) is a class I transmembrane receptor expressed on the surface of immune system cells. TLR4 is activated by exposure to lipopolysaccharides derived from the outer membrane of Gram negative bacteria and forms part of the innate immune response in mammals. Like other class 1 receptors, TLR4 is activated by ligand induced dimerization, and recent studies suggest that this causes concerted conformational changes in the receptor leading to self association of the cytoplasmic Toll/Interleukin 1 receptor (TIR) signalling domain. This homodimerization event is proposed to provide a new scaffold that is able to bind downstream signalling adaptor proteins. TLR4 uses two different sets of adaptors; TRAM and TRIF, and Mal and MyD88. These adaptor pairs couple two distinct signalling pathways leading to the activation of interferon response factor 3 (IRF-3) and nuclear factor κB (NFκB) respectively. In this paper we have generated a structural model of the TLR4 TIR dimer and used molecular docking to probe for potential sites of interaction between the receptor homodimer and the adaptor molecules. Remarkably, both the Mal and TRAM adaptors are strongly predicted to bind at two symmetry-related sites at the homodimer interface. This model of TLR4 activation is supported by extensive functional studies involving site directed mutagenesis, inhibition by cell permeable peptides and stable protein phosphorylation of receptor and adaptor TIR domains. Our results also suggest a molecular mechanism for two recent findings, the caspase 1 dependence of Mal signalling and the protective effects conferred by the Mal polymorphism Ser180Leu.

## Introduction

In humans and other vertebrates initial responses to infection by pathogenic microorganisms such as viruses and bacteria are mediated by a highly developed innate immune response[1]. Pattern recognition receptors (PRRs) expressed by immune system cells such as macrophages and dendritic cells are able to detect conserved microbial structures. These cells then generate the innate immune responses that are required to fight the infection and promote the development of adaptive immunity. The Toll-like receptors (TLRs) are an important group of PRRs that respond to a range of microbial products such as lipopeptides and non-self nucleic acids [2]. The TLRs are type I transmembrane receptors and consist of an extracellular domain made up mainly of leucine rich repeat motifs, a single transmembrane spanning segment and a globular cytoplasmic domain, the Toll/interleukin 1 receptor domain (TIR) [3]. There are ten Toll-like receptors encoded in the human genome and each of these respond to specific microbial products.

One of the most important innate immune stimuli is lipopolysaccharide (LPS) or endotoxin found in the outer membrane of Gram-negative bacteria [4]. LPS is one of the most powerful immunostimulators known and is responsible not only for the induction of innate immunity but also for the dangerous condition endotoxic shock which often develops during Gram negative septicaemia. Endotoxic shock is a severe inflammatory disease that leads rapidly to multi organ failure and death. This condition accounts for about 200,000 deaths per annum in Europe and thus understanding the mechanism of action of LPS mediated immune activation is an important objective in medical research [5]. In 1998 TLR4 was identified as the signalling receptor for LPS. Mice that lack functional TLR4 are hyposensitive to LPS and consequently more sensitive to infection by Gram negative bacteria [6]. Subsequent studies showed that MD-2, a co-receptor protein of TLR4, was also essential for LPS induced signalling [7], [8]. MD-2 is a member of a small class of lipid binding proteins and interacts directly with the lipid A moiety of LPS [9], [10].

**Figure 1:**
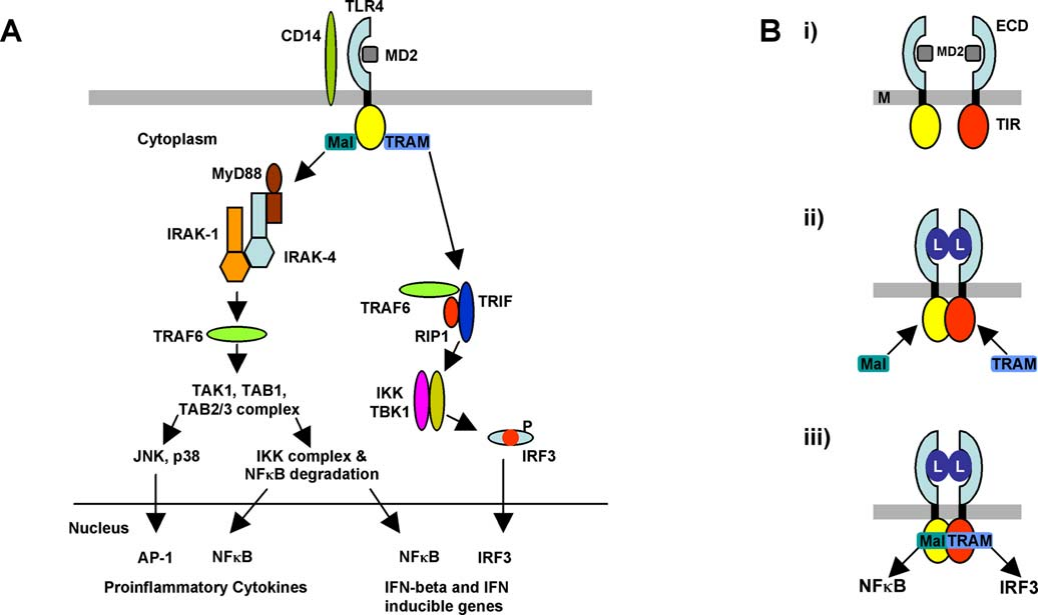
The TLR4 signalling pathway. (A) Overview of the TLR4 signalling pathway. Both the NF-κB and the interferon pathways are induced by stimulation with lipopolysaccharide. Adapted from [2], [3]. (B) Mechanism of signal transduction by TLR4. The curved ectodomains (ECD) are illustrated in light blue and the co-receptor protein MD-2 in grey. The TIR domains are shown in yellow and red respectively. M = membrane, L = LPS. (i) Prior to activation, receptor molecules are able to diffuse in the membrane and may form transient dimers. The ectodomains are rigidly connected to the cytoplasmic TIRs by the transmembrane helix. (ii) Receptor dimerization following activation by LPS binding to MD2. By analogy with *Drosophila* Toll (see [45]), which is activated by a dimeric protein ligand, the receptor complexes are likely to be symmetrical. Conformational rearrangements constrain the TIR domains to interact through equivalent surfaces forming a symmetrical dimer. (iii) The dimerized TIRs provide a new molecular surface that can bind to the ‘bridging adaptor’ molecules TRAM and Mal with high affinity. Interaction with the downstream adaptors TRIF and MyD88 leads to NFκB and IRF3 mediated signalling respectively.

Like other class I receptors the initial step in signal transduction by TLR4 involves dimerization or oligomerization of two receptor chains induced by binding of MD-2 to the lipid A moiety of LPS [11]. This in turn probably causes protein conformational changes in the receptor resulting in the association of two receptor TIR domains (Figure 1) [12]. Alternatively, the receptor may be present in the cell as a preformed but inactive dimer and ligand binding may cause reorientation of the TIR domains. Consistent with this idea, a recent study using FRET (fluorescence resonance energy transfer) microscopy showed that the TLR9 TIR domains undergo a large positional change on ligand binding [13]. In either case association of the receptor TIRs would provide a new scaffold that allows the recruitment of specific adaptor proteins to form a post-receptor signalling complex. There are five adaptor proteins, all of which contain TIR domains, that function in TLR signalling; MyD88 (Myeloid differentiation primary response protein 88), Mal (MyD88 adaptor like; also known as TIRAP), TRIF (TIR domain-containing adaptor protein inducing interferon-β; also known as TICAM1), TRAM (TRIF-related adaptor molecule; also known as TICAM2), and SARM (sterile α- and armadillo-motif-containing protein) [14]. The TIR domain forms into an α−β structure and the sequence conservation observed reflects the structural requirements of this fold [15]. On the other hand, the loops that connect the secondary structure elements of the TIR domain and the surface electrostatic properties are more variable and these properties may confer specificity for homo- and heterotypic interactions between different TIR domains [16].

Unlike the other TLRs, activated TLR4 signals via two distinct sets of adaptor proteins, Mal and MyD88, and TRAM and TRIF [17], [18] (see Figure 1). For each pathway Mal and TRAM are thought to engage directly with the TLR4 receptor dimer and subsequently act as ‘bridging adaptors’ for the recruitment of MyD88 and TRIF respectively. Mal is required for rapid activation of the ΝFκΒ transcription factor and the production of pro-inflammatory cytokines such as TNFα and IL12. The TRAM adaptor on the other hand stimulates a different pathway leading to activation of the interferon response factor, IRF-3. IRF-3 induces expression of a distinct set of genes to NFκB, such as interferon β and the chemokine RANTES. It is not known whether a single activated TLR4 dimer can recruit both TRAM and Mal simultaneously or whether binding is mutually exclusive.

In this paper we have used modelling, docking and mutagenesis studies to investigate the nature of the initial TLR4 receptor dimer-‘bridging adaptor’ complexes. We show that both Mal and TRAM are strongly predicted to bind at the interface of a TLR4 TIR homodimer, a result consistent with multiple other studies using mutagenesis of receptor and adaptor TIRs and blocking peptides.

## Results

### Predicted structure of the TLR4 TIR domain homodimer and the Mal and TRAM adaptors

The structure of the receptor TIR domains from TLR1 and 2 was solved previously by X-ray crystallography [19]. These modules behave as monomers in solution and the packing of the molecules in the crystal lattice does not suggest a likely arrangement for the functional homodimer generated during receptor activation. More recently in the course of a structural genomics project, the TIR domain of TLR10 has been solved (http://sgc.utoronto.ca/SGC-WebPages/StructureDescription/2J67.php). In contrast to TLR1 and 2, the TLR10 TIR domain is present as a dimer in the asymmetric unit. The molecules are related by a two-fold axis of symmetry and there is an extensive dimer interface. In addition, the region of the TIR domain involved in dimer formation includes a surface structural element called the BB-loop that links the second β-sheet to the second α-helix. The BB loop is thought to be important for homodimer formation because mutations of a conserved proline residue inactivate signalling and exert dominant negative effects, indicating the formation of non-functional heterodimers of the wild-type and mutant receptor chain. Thus it is likely that the TLR10 TIR structure represents a physiologically relevant conformation.

**Figure 2:**
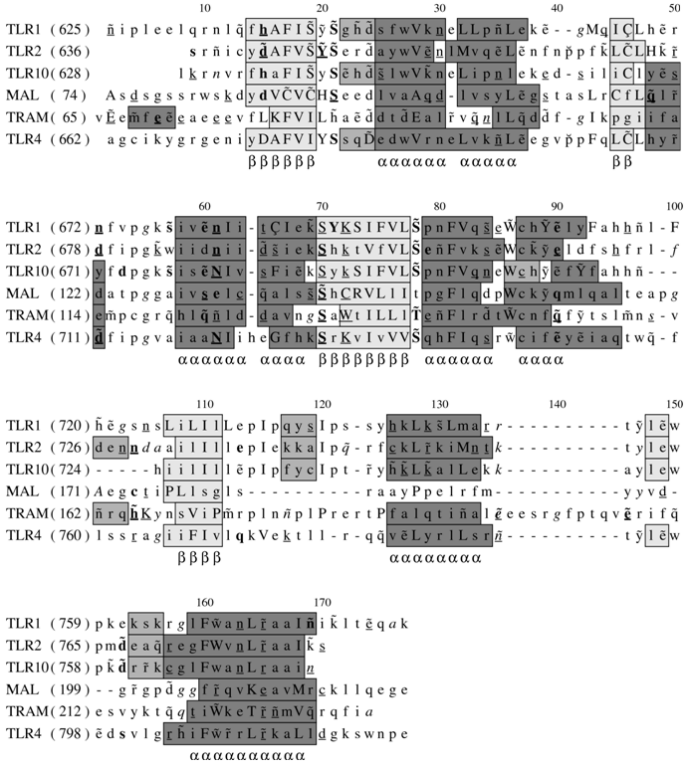
Structure based sequence alignments of TIR domains. The program JOY was used to annotate the alignments for TLR1, TLR2 TLR4, TLR10, Mal and TRAM. Numbers on top of amino acid sequences are alignment positions. The key to JOY annotations is as follows (a graphical version is viewable as Table S1); solvent inaccessible – UPPER CASE; solvent accessible – lower case; α-helix – dark grey shaded; β-strand – mid-grey shaded; 3_10_ helix – light grey shaded; hydrogen bond to main chain amide – bold; hydrogen bond to main chain carbonyl – underline; hydrogen bond to other sidechain – tilde; disulphide bond – cedilla; positive φ--->φ - *italic*; *cis*-peptide – breve.

**Figure 3:**
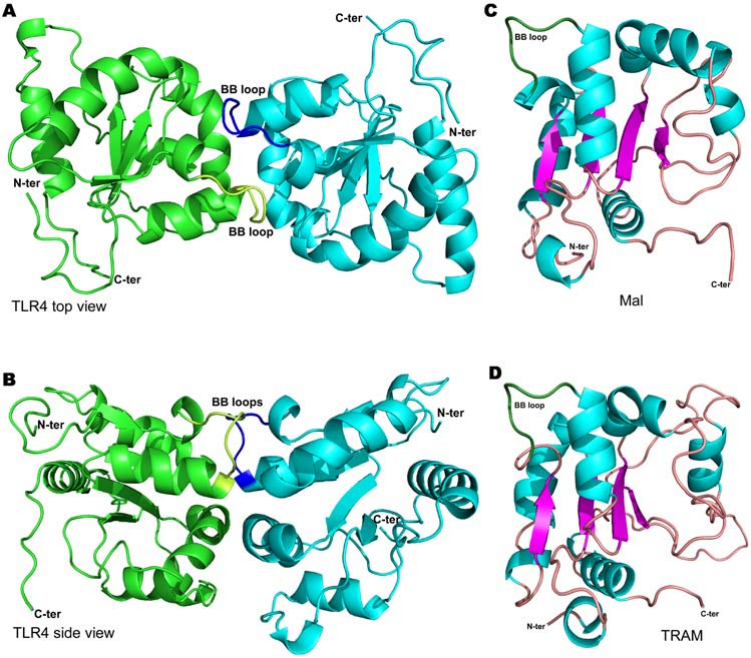
Structural modelling of the TLR4 TIR domain homodimer, Mal and TRAM. The BB loops of the two TLR4 protomers are coloured blue and yellow respectively. For Mal and TRAM they are coloured green (A) TLR4 top view. (B) TLR4 side view. (C) Mal. (D) TRAM.

In light of this, and the widely held view that signal induced dimerization of the receptor cytoplasmic domains is required for signalling [12], we decided to model the TLR4 TIR domain as a homodimer, reasoning that this structure would approximate the configuration adopted in the activated receptor complex. This approach allows significant advances to be made in the interpretation of our modelling results over previous work in which the TLR4 TIR was modelled as a monomer [16]. The crystal structures of TLR1 and TLR2 (Pdb: 1FYV and 1FYW) together with the recently released crystal structure of the human TLR10 dimer (Pdb: 2J67) were used as templates to build the TLR4 structural model (Figure 2). In the alignments used for the modelling sequence identity between TLR4 and TLR1, TLR2 and TLR10 was 35.0%, 40.5% and 35.0%, respectively. The secondary structure of the modelled TLR4 TIR domain is similar to that of the templates. It is predicted to contain five β strands and six α helices, with the BB loop adopting a similar conformation to that in the template structures (Figures 2, 3A and 3B). Analysis of the structural model of the TLR4 dimer reveals that 94.2% of the phi-psi dihedral angles are found in the most favourable regions of the Ramachandran plot. The remaining 5.8% are found within the allowed regions. This highlights the excellent geometry of the model. Verify3D [20] reports no values below 0.21, further indicating that all the residues are located in favourable structural environments. The JOY output also shows that the residues in the model are in environments similar to those of the templates (Figure 2).

The surface area buried at the dimer interface of the TLR4 homodimer has contributions of 657 Å^2^ and 665 Å^2^ from each of the two protomers. Table 1 lists the 18 residues that make van der Waals contacts with residues from the other chain in the dimer interface. Interestingly, some interactions are non-reciprocal. For example, Arg745 from chain A makes an interaction that buries 42.4 Å^2^ of surface but the corresponding residue in chain B buries only 8.6 Å^2^. Similarly, Arg780 from chain A buries a surface of 30.8 Å^2^ whilst the same residue from chain B does not interact. Weak inter-subunit electrostatic interactions are also observed. The strongest is between Glu750 from one chain and Arg780 from the other. However, the distance between charged atoms is 9.6 Å. Significantly the BB loops from the two protomers also interact with each other. In particular, the residue Phe712 from each subunit forms an aromatic interaction with one another. Interestingly, the dimer has a flat but slightly curved surface (Figure 3B – side view) which may be the top (or membrane proximal) surface of the structure (see discussion).

**Table 1:** Interacting residues in the dimer interface of TLR4.

Residue Number	Residue type	Chain	ΔASA (Å^2^)	Residue Number	Residue type	Chain	ΔASA (Å^2^)
682	Ser	A	16.3	682	Ser	B	9.5
708	His	A	19.6	708	His	B	31.8
709	Tyr	A	88.5	709	Tyr	B	75.0
712	Phe	A	50.8	712	Phe	B	45.0
714	Pro	A	79.0	714	Pro	B	79.4
715	Gly	A	18.5	715	Gly	B	15.4
716	Val	A	7.0	716	Val	B	8.2
717	Ala	A	35.4	717	Ala	B	50,2
718	Ile	A	99.3	718	Ile	B	99.6
719	Ala	A	15.0	719	Ala	B	23.1
743	Gln	A	11.4	743	Gln	B	5.7
744	Ser	A	14.2	744	Ser	B	31.5
745	Arg	A	42.4	745	Arg	B	8.6
747	Cys	A	55.8	747	Cys	B	71.0
748	Ile	A	27.9	748	Ile	B	33.8
750	Glu	A	1.2	---	---	-	---
751	Tyr	A	42.9	751	Tyr	B	33.9
---	---	-	---	754	Ala	B	4.9
---	---	-	---	755	Gln	B	37.2
780	Arg	A	30.8				

We have also generated high quality models of the Mal and TRAM adaptor TIR domains (Figure 3C, D), using the crystal structures of TLR1 and 2 as templates (Figure 2). In the alignments used Mal displays amino acid sequence identity with TLR1 and TLR2 of 21.1% and 23.0%, respectively. As expected the secondary structure of the Mal TIR domain is similar to that of the templates with the exception of a nine amino acid deletion before the start of the 4^th^ helical segment (alignment position 114 to 122 in Figure 2). This deletion results in structural distortion and loss of the αD helix and a small β strand. The model of the Mal TIR domain also has good geometry. None of its phi-psi dihedral angles are found within disallowed regions of the Ramachandran plot, whilst 90.6% are in geometrically favoured regions. The JOY and Verify3D outputs show that the environments of residues in the model of Mal are similar to those of the templates and are not energetically unfavourable (Figure 2). Overall the structure of Mal is a four-stranded β sheet surrounded by five α helices in which the BB loop adopts a fixed conformation similar to that seen in TLR1 and 2. A similar strategy was used to model the structure of the TRAM adaptor. In this instance the amino acid sequence identity between the modelled regions of TRAM and TLR1 and TLR2 are 15.5% and 15.9%, respectively. With the exception of a ten amino acid insertion before the last small β strand (alignment position 136 to 145 in Figure 2) the secondary structure of the TRAM TIR domain is similar to that of the templates. The phi-psi dihedral angles of the TRAM model show good geometry. The Ramachandran plot contains 88.5% in the most favourable regions, 10.9% in allowed regions and only a single residue (0.6%) in disallowed regions. As with Mal and TLR4 the residues in the TRAM model are predicted to be in structurally favourable environments.

### The MAL and TRAM adaptors are predicted to bind at the TLR4 dimer interface

**Figure 4:**
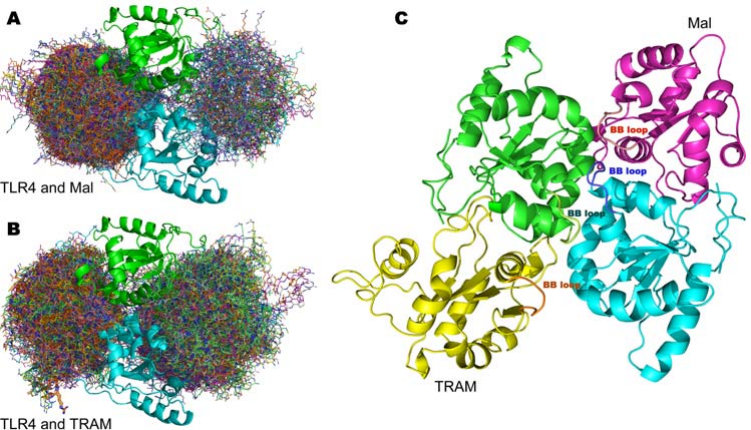
Docking studies predict that the adaptors bind at the side of the TLR4 homodimer interface. The TLR4 protomers, represented as ribbon diagrams are in green and cyan. Docked Mal and TRAM are represented as stick models and the 50 best docking solutions generated by GRAMM for either Mal (A) or TRAM (B) have been superimposed upon one another. (C) High resolution complex of TLR4 dimer (green and cyan), Mal (pink) and TRAM (yellow). The position of each BB loop is labelled.

We have used GRAMM [21], a molecular docking programme that uses shape complementarity to assess the interaction of protein molecules, to probe the likely binding sites in the TLR4 dimer for the adaptors Mal and TRAM (Figure 4A, B). Consideration of TLR4 as a dimer creates a more physiologically relevant receptor docking template compared with earlier studies in which the TLR4 receptor was modelled as a monomer [16]. Remarkably, the 100 best low resolution docking solutions for Mal lie at the TLR4 dimer interface indicating that dimer formation creates two specific symmetry related binding sites for this molecule. The TRAM adaptor is predicted to bind to the same site as Mal. The interaction surfaces on the TLR4 dimer interface are at either side of the structure rather than at the top, a region that would be sterically hindered by the membrane.

We then carried out high resolution docking experiments using the programme PyDock [22]. These experiments were guided through the use of new, and previously published, information from mutagenesis studies. In particular we took account of mutants located on the side surface of the dimer (His728Asp, Trp757Ala, Gln683Ala, IleIle723-724AlaAla, His724Ala, GlyPhe726-728AlaAla, Gln758Ala, Arg763Ala; see Table 2, 3; and ref [23]). We also considered the impact of reported contributions from Mal (BB loop residues and a tyrosine phosphorylation site, Tyr86, located close to the BB loop, see discussion). The high resolution model of the TLR4 homodimer with Mal and TRAM bound is shown in Figure 4C with Mal in magenta and TRAM in yellow.

**Table 2:** Potential structural impact of TLR4 TIR mutations.

Mutation	Structural justification for functional impact
YD674-675AA	Loss of hydrogen bonding - structural destabilisation
FVI677-679AAA	Buried – structural disruption of hydrophobic core
YSS680-682AAA	Disruption of TLR4 TIR dimer formation
Q683A	Possible interference with adaptor binding
K694A	Disrupt favourable electrostatic interactions
NLE695-697AAA	Disrupt favourable electrostatic interactions
EG698-699AA	Possible interference with adaptor binding
VP700-701AA	Possible interference with adaptor binding
C706S*	Buried – structural disruption of hydrophobic core
LCL705-707AAA	Buried – structural disruption of hydrophobic core (poor expression)
HYR708-710AAA	Disrupt favourable electrostatic interactions and dimerization
DFI711-713AAA	Loss of hydrogen bonding, loss of favourable electrostatic interactions, disruption of dimer formation
P714H*	Structural distortion and disruption of dimer formation
PGV714-716AAA	Disruption of TLR4 TIR dimer formation
I718A	Disruption of TLR4 TIR dimer formation
II722-723AA	Possible interference with adaptor binding
H724A	Possible interference with adaptor binding
E725A	Disrupt favourable electrostatic interactions
G726C*	Buried – structural disruption of hydrophobic core
GF726-727AA	Buried – structural disruption of hydrophobic core
H728D*	Possible interference with adaptor binding
K729A	Disrupt favourable electrostatic interactions
VIV733-735AAA	Buried – structural disruption of hydrophobic core
VVS736-738AAA	Buried – structural disruption of hydrophobic core
QH739-740AA	Loss of hydrogen bonding - structural destabilisation
IQ742-743AA	Loss of hydrogen bonding - structural destabilisation
SR744-745AA	Disruption of TLR4 TIR dimer formation
C747S*	Disruption of TLR4 TIR dimer formation
YE751-752AA	Disruption of TLR4 TIR dimer formation
I753A	Possible interference with adaptor binding
Q755A	Disruption of TLR4 TIR dimer formation
TW756-757AA	Loss of hydrogen bonding - structural destabilisation
Q758A	Possible interference with adaptor binding
FL759-760AA	Possible interference with adaptor binding
R763A	Possible interference with adaptor binding
GI765-766AA	Buried – structural disruption of hydrophobic core
IFI767-769AAA	Buried – structural disruption of hydrophobic core
K773A	Disrupt favourable electrostatic interactions
EK775-776AA	Possible interference with adaptor binding
QQ781-782AA	Loss of hydrogen bonding – structural destabilisation
RL787-788AA	Possible interference with adaptor binding
TY793-794AA	Possible interference with adaptor binding
EWE796-798AA	Disrupt favourable electrostatic interactions
DS799-800AA	Reduction in expression levels
G803A	Possible interference with adaptor binding
HI805-806AA	Possible interference with adaptor binding
FWR807-809AAA	Possible interference with adaptor binding
RR809-810AA	Possible interference with adaptor binding
RLR810-812AAA	Possible interference with adaptor binding
LR811-812AA	Possible interference with adaptor binding
L815A	Buried – structural disruption of hydrophobic core
Y794STOP	Disruption of electrostatic surface and possible interference with adaptor binding
R809STOP	Poor expression
W821STOP	Possible interference with adaptor binding

Mutants displaying <75% wild-type activity in NFκB reporter assays were interpreted for their potential impact on TLR4 TIR structure and used to guide docking studies. Data obtained from Ronni et al [23] except for those marked with an asterisk which were assayed as part of this study (see also Table 3).

**Table 3:** Summary of effects of mutation on NFκB and IFN-β activation.

TLR4 Mutation	NFκB activation	IFN-β activation
E698K	S	S
C706S	RS	nd
P714H	NS	NS
G726C	NS	nd
H728D	RS	RS
Q743A	S	S
C747S	RS	nd
W757A	S	RS
K776D	S	S
C706S/C747S	NS	nd

All mutations were tested in at least three independent assays. S = signals comparative to wild-type, RS = reduced signal compared to wild-type, NS = no signalling, nd = not determined.

The buried surface at the interface of the TLR4 dimer-Mal complex constitutes 1824 Å^2^ from the TLR4 dimer and 1761 Å^2^ from Mal. There are 17 residues from TLR4 chain A and 28 residues from chain B that make contacts (ΔASA>1 Å^2^) with 49 residues from Mal. Table 4 shows those residues that produce the strongest van der Waals interactions (ΔASA>40 Å^2^) in the complex interface. For TLR4 this is Trp757, and for Mal Glu167. Seven hydrogen bonds are present in the interface of the TLR4 dimer-Mal complex, donated by three residues from each TLR4 chain and six residues from Mal. This includes a double hydrogen bond between TLR4 His728 and Mal Thr166. Strong electrostatic interactions are present between charged residues from both components of the complex. The strongest salt bridge is between the TLR4 Arg763 and Mal Glu167. Also important is the ion pair between TLR4 Lys819 and Mal Glu167.

**Table 4:** Residues that produce strong interactions (ΔASA>40 Å^2^) in the interface of the TLR4 dimer-Mal complex.

Residue Number	Residue type	TLR4 Chain	ΔASA (Å^2^)	Residue Number	Residue type	Mal Chain	ΔASA (Å^2^)
683	Gln	A	91.3	78	Gly	C	65.3
685	Glu	A	48.9	81	Arg	C	74.0
740	His	A	59.6	82	Trp	C	57.4
743	Gln	A	76.7	83	Ser	C	111.9
778	Leu	A	126.1	85	Asp	C	84.0
780	Arg	A	53.2	124	Thr	C	62.9
669	Arg	B	46.5	130	Val	C	41.8
671	Glu	B	85.9	131	Ser	C	41.2
724	His	B	116.0	134	Cys	C	42.9
728	His	B	88.3	135	Gln	C	95.0
755	Gln	B	69.3	138	Ser	C	52.5
757	Trp	B	175.2	161	Met	C	46.9
824	Glu	B	161.4	165	Leu	C	116.0
				166	Thr	C	65.5
				167	Glu	C	122.2
				168	Ala	C	58.9
				172	Glu	C	80.7
				187	Tyr	C	53.5
				221	Glu	C	46.7

The interface of the TLR4 dimer-TRAM complex results in a buried surface of 1729 Å^2^ from the TLR4 dimer and 1805 Å^2^ from TRAM. TLR4 chain A and B contribute 33 and 9 residues respectively, which form contacts (ΔASA>1 Å^2^) with 43 residues from TRAM. Table 5 shows those residues that produce strong van der Waals interactions (ΔASA>40 Å^2^) in the complex interface. As with the Mal complex, TLR4 Trp757 makes the strongest interaction with TRAM, whilst TRAM Asn 159 interacts most strongly with TLR4. In this complex only three hydrogen bonds are present in the interface. TLR4 chain A donates three residues and TRAM two, of which one, Asp164, produces two strong hydrogen bonds. Strong electrostatic interactions are observed between charged residues from both components of the complex. Salt bridges are formed between TLR4 Glu824 and TRAM Arg231 and TLR4 Glu685 and TRAM Arg119. Also important is the ion pair between TLR4 Arg780 and TRAM Glu75.

**Table 5:** Residues that produce strong interactions (ΔASA>40 Å^2^) in the interface of the TLR4 dimer-TRAM complex.

Residue Number	Residue type	TLR4 Chain	ΔASA (Å^2^)	Residue Number	Residue type	TRAM Chain	ΔASA (Å^2^)
669	Arg	A	108.3	73	Ala	D	48.3
671	Glu	A	77.7	74	Glu	D	101.0
724	His	A	101.9	75	Glu	D	133.7
728	His	A	106.2	119	Arg	D	52.8
755	Gln	A	130.1	130	Asn	D	73.5
757	Trp	A	155.7	153	Phe	D	86.3
758	Gln	A	43.4	155	Thr	D	74.7
760	Leu	A	78.1	156	Ser	D	107.1
819	Lys	A	60.1	157	Leu	D	49.3
821	Trp	A	50.8	159	Asn	D	152.1
822	Asn	A	90.3	160	Ser	D	51.0
824	Glu	A	68.0	163	Arg	D	147.9
743	Gln	B	90.1	164	Gln	D	118.6
780	Arg	B	123.7	233	Phe	D	71.7
				235	Ala	D	45.3

We next used the FPSPD program (see Methods) to predict functional sites in TLR4, Mal and TRAM. The patterns found for TLR4 were: residues 677–698, 705–730, 734–759 and 795–815. The first functional pattern 677–698 contains residues involved in Mal binding (Table 4). Pattern 705–730, which includes the BB loop, possesses residues for homodimerization, and TRAM binding (Tables 1 and 5) and pattern 734–759 is implicated in homodimerization and Mal binding (Tables 1 and 4). Finally, pattern 795–815 does not contribute to any of the interactions identified in this study but may represent a binding site for a second adaptor molecule such as MyD88. In the case of Mal, the functional patterns predicted by FPSPD are: residues 89–104, 108–122, 121–139 and 146–169. All these are directly involved in TLR4 binding except pattern 108–122. However, this one is spatially close to the TLR4 pattern 795–815 in the TLR4 dimer-Mal complex. Thus these patterns could both contribute to a binding site that is only formed after initial recruitment of Mal. In the case of TRAM the patterns identified are: residues 72–94, 117–127, 136–164, 171–181 and 218–226. Patterns 72–94 and 136–164 have involvement in TLR4 binding, see (Table 5). Only one residue from pattern 117–127 interacts with TLR4, and neither pattern 171–181 nor 218–226 contain residues that interact with TLR4. These regions may potentially be involved in binding of a second adaptor, for example TRIF. Interestingly, pattern 117–127 is spatially equivalent to the Mal pattern 108–122.

In conclusion, this modelling study predicts that homodimerization of the TLR4 TIR domain creates a scaffold for the recruitment of adaptor molecules into a post receptor complex. Remarkably the study predicts that the ‘BB’ loop structures of all three molecules are critical determinants of binding specificity (Figure 4C).

### Functional studies of the TLR4, MAL and TRAM TIR domains support the proposed mechanism of receptor activation

Although experimental structural analysis of TLR4 has proved difficult a number of studies have identified regions of the receptor and adaptor TIRs that are important for signalling function. In particular these studies have utilised mutagenesis, the use of cell permeable blocking peptides and studies of Mal tyrosine phosphorylation.

Table 2 summarises the results of mutagenesis studies on the TIR domain of TLR4 and the effect that the mutations are predicted to have on the receptor-adaptor complexes. This includes previously unpublished data together with the results reported by Ronni et al [23]. In general the effect of mutating individual residues is variable, ranging from a substantial loss of function, such as the BB loop proline residue, to partial or no loss of signalling. Our analysis indicates that the mutants that impair function can be grouped into three classes. The first are those such as P714 that are likely to impair receptor homodimerization or cause the formation of non-functional receptor dimers that are not able to bind adaptors. These residues predominantly lie in two discrete regions of the molecule, residues 708–718, containing the BB-loop, and residues 744–755 which form the αC helix/CD loop. The second group are residues that lie in the putative adaptor binding surface created by the dimer interface. The modelling study identifies two other surfaces that may be interaction sites with adaptors or other protein factors involved in TLR4 signalling, one on the sides of the dimer and one on the bottom. These regions may represent secondary sites of interaction for MyD88 and TRIF and interestingly a previous study pointed to the CD loop located on the bottom surface as a binding site for MyD88 [16]. The third group of mutants are those that make important intra-chain contacts and are probably required for the stability of the TIR domain fold. We have also assayed a number of mutant receptors for signalling to both NFκB (a Mal directed target) and IFNβ (a TRAM target). The results (Table 3) show that mutations on the whole have similar effects on both arms of the TLR4 pathway consistent with the Mal and TRAM adaptors binding to the same or overlapping site on the activated receptor.

In addition to mutagenesis, studies with cell permeable blocking peptides also provide support for the model proposed here. Peptides corresponding to the BB-loop of TLR4 strongly inhibit LPS induced responses mediated by both the TRAM and the Mal adaptors [24]. According to our model this peptide would compete with the BB loops in the receptor TIRs and prevent or disrupt the formation of the homodimer. BB-loop peptides from Mal and TRAM also inhibit LPS signalling, again affecting both arms of the pathway [25]. This result is also consistent with our model as it provides strong evidence that the BB-loops of Mal and TRAM play a critical role in binding to the activated receptor. As the peptides block both NFκB and IRF-3 directed responses this result also indicated that the binding sites for the two adaptors overlap. The same study also found that a BB-loop peptide from MyD88 inhibited signalling by LPS. This finding suggests that initial binding of the Mal or TRAM adaptors creates a new surface for association of MyD88 and TRIF with determinants contributed by both the receptor and the adaptor TIRs, possibly involving the bottom surface of the homodimer.

**Figure 5:**
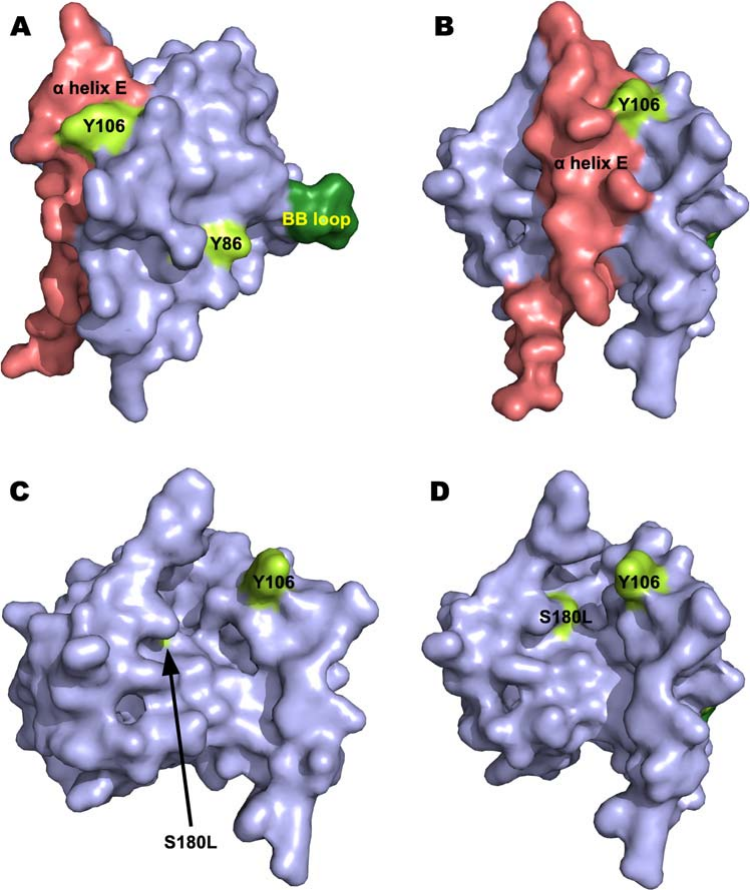
Modelling suggests a molecular explanation for the caspase 1 dependence of Mal and the malfunctional human polymorphism Ser180Leu. The models are shown as van der Waal surface representations. (A) Side view showing the position of the BB loop (green) and the phosphorylated tyrosine, Tyr86. In the complex this part of Mal forms the interface with TLR4. The position of the α-E helix (red) which is cleaved out by caspase 1 is shown on the opposite surface to the BB loop. (B) Back view of Mal (rotated 90° to the right relative to (A)). (C), (D) Mal with the α-E helix removed highlighting the deep groove created and the exposed position of the otherwise buried Ser180 residue (yellow).

A third line of evidence in support of the model comes from studies of tyrosine phosphorylation. Mal is phosphorylated by the Bruton tyrosine kinase at positions 86, 106 and 187 and substitution of tyrosine with phenylalanine at positions 86 and 187 impairs LPS mediated signalling to NFκB [26]. As shown in Figure 5, Tyr86 is located close to the BB-loop and should interfere with binding of Mal to TLR4. In our complex Tyr86 makes an interaction that buries 33.0 Å^2^ of surface. Interestingly, co-immunoprecipitation experiments indicate that the phospho forms of Mal do not bind to TLR4.

## Discussion

The structural modelling and functional studies presented here provide strong support for theoretical models of signal transduction by TLR4 and other Toll family receptors [12]. In this view, stimulus induced dimerization of the receptor extracellular domains leads to concerted protein conformational changes that in turn lead to self-association or rearrangement of the receptor TIRs thereby creating a new molecular surface for the recruitment of signalling adaptor proteins. In our model of the TLR4 homodimer the interface has extensive interactions involving the BB loops of the two subunits. The conserved proline residue will confer a rigid conformation on the BB-loop and substitution by other residues would cause considerable distortion in the geometry of the homodimer interface. Another important conclusion of this study is that the receptor TIRs associate with a 2-fold axis of symmetry. This implies that the linkers between the membrane and the TIR domains have rotational flexibility. This seems plausible as the linkers are fairly long (about 20 amino acids) and contain glycine residues that can adopt a wider range of dihedral angles than the chiral amino acids. Another interesting feature of the TLR4 dimer is the flat but slightly curved surface predicted to form the top or membrane proximal surface of the structure (Figure 3B). This architecture is seen in other proteins that interact with membrane surfaces, for example the BAR domain of amphiphysin [27].

Our model also predicts that Mal and TRAM bind to the same region in the TLR4 dimer interface. This explains why cell permeable blocking peptides compete out both Mal and TRAM directed responses simultaneously [24], [25]. However, the model does not resolve the question of whether a single activated receptor dimer can stimulate both the Mal and TRAM directed pathways simultaneously or whether adaptor engagement is mutually exclusive, something that would require positive cooperativity. Whilst we are currently addressing this question, it is clear however, that each activated receptor will have two symmetry related adaptor binding sites so in principle either hypothesis is feasible.

Previous studies have attempted to model the interactions of Mal and MyD88 with TLR4 and TLR2 [16]. However, these used monomeric receptors in the modelling process. It is now widely believed that receptor activation leads to ligand induced dimerization [12]. Consequently our current studies have modelled the activated TLR4 receptor as a dimer and are therefore likely to be more physiologically relevant. This may explain the altered orientation of Mal binding compared to the previous study. Our observations are however broadly consistent with the earlier predicted location of a binding site for MyD88 on TLR4. Further, our work suggests that initial binding of the ‘bridging adaptor’ proteins may contribute to formation of an additional surface for secondary adaptor binding.

The predicted adaptor/receptor binding surface is extensive and is contributed to by many residues from both the receptor and the adaptors. This may explain why many mutations in the receptor TIR only partially impair signalling (see Tables 2 and 3 and ref [23]). Changing a single favourable receptor-adaptor contact might result in a decrease in affinity of the complex but would not abolish function completely.

The docking results suggest that Mal binds the TLR4 homodimer with a closer proximity and greater number of direct contacts than TRAM does (Tables 4 and 5). This may suggest a higher affinity for Mal binding and would perhaps explain why TRAM is not subjected to such high levels of cellular regulation and also why Mal/MyD88 pathways trigger early NF-κB expression, but TRAM/TRIF signalling results in late NF-κB expression. Moreover, phosphorylation by Bruton tyrosine kinase of Mal at Tyr86, a residue predicted to interact strongly with the TLR4 homodimer may render Mal-TLR4 interaction sterically unfeasible in addition to facilitating SOCS-1 mediated Mal polyubiquitination and degradation [28]. Interestingly, the equivalent residue in TRAM is Phe78 suggesting that the binding between TRAM and the receptor dimer may be a relatively weaker hydrophobic interaction.

Two other recently published results concerning Mal are also of interest in the context of this model. Firstly, Miggin et al. [29] have shown that Mal is cleaved at position 198 by the cysteine protease caspase 1 and that this processing is required for Mal to function in signalling by both TLR2 and TLR4. The cleavage releases a 4 kDa fragment from the C-terminus of Mal (see Figure 2 and 5) corresponding to the ‘EE’ loop and the last (E) α-helix of the TIR fold. As shown in Figure 5, the site of proteolysis is on the surface opposite the BB-loop and thus should not affect binding of Mal to the receptor dimer. One explanation for this finding might be that in the absence of caspase cleavage MyD88 cannot be recruited into the postreceptor complex by Mal. Plausibly MyD88 might displace the ‘E’ helix from Mal and bind into the cleft that is exposed (Figure 5A–D). The second study concerns a variant of Mal, Ser180Leu, which is common in the human population. When heterozygous this polymorphism protects against a range of microbial infections and cell based assays show that the mutation significantly impairs signalling by both TLR2 and TLR4 [30]. In our model of Mal this residue is buried in the structure but would be exposed after cleavage with caspase 1 (Figure 5C, D). Thus it is possible that the observed loss of function associated with Ser180Leu is caused by a defect in recruitment of MyD88 to caspase-1 cleaved Mal. With the replacement of serine by a large, hydrophobic leucine residue introducing an unfavourable interaction in the caspase-1 cleaved adaptor.

In conclusion, the current study provides a basis for future structural and functional studies of TLR4 activation. A long term objective is to carry out experimental structural analysis of activated membrane receptor complexes of TLR4 using low resolution techniques such image reconstruction of electron micrographs as well as protein crystallography.

## Materials and Methods

### Molecular modelling

The amino acid sequences and three dimensional structures of the homologous proteins used as templates for comparative modelling were obtained from the Protein Data Bank (http://www.rcsb.org/pdb). Initial alignments between target protein and its templates were obtained using the program FUGUE [31]. FUGUE produces alignments by comparison of sequence profiles with structural profiles of homologous protein families taken from the HOMSTRAD database [32].

Models were produced using the program MODELLER [33]. MODELLER produces comparative models satisfying spatial restraints with simultaneous optimization of CHARMM energies [34]. This method uses conjugate gradients and molecular dynamics with simulated annealing [33]. The form of the spatial restraints was obtained by statistical analysis of the relationship between pairs of homologous structures from a database of sequence alignments for 416 proteins of known 3D structure in 105 families. Comparative models were verified by the validation programs PROCHECK [35], VERIFY3D [20] and JOY [36]. The alignments were then manually modified as required and the modelling and validation process repeated. The process of modelling, validation and realignment was repeated until models with good geometry and conformation had been obtained.

Contact residues in the TLR4 homodimer and docked complexes were defined as the residues that possessed an interface solvent accessible surface area (ASA) that decreased (ΔASA) by more than 1 Å^2^ on complexation [37]. The ASA was calculated using the Lee and Richards algorithm [38] developed by Richmond [39]. HBPLUS was used for hydrogen bond definition [40]. Electrostatic interactions were calculated using an in house program (ELECINT, R.N. Miguel, unpublished) using a dielectric constant distance dependent for electrostatic field calculation and pH = 7.4 for the calculation of charges of side chain atoms of charged residues using the Henderson-Hasselbalch equation.

### Protein docking and binding site prediction

Low resolution protein-protein docking was carried out using Global Range Molecular Matching (GRAMM) methodology [41]. GRAMM methodology is an empirical approach to smoothing the intermolecular energy function by changing the range of the atom-atom potentials. Low resolution docking is useful for determining possible relative positions of the two proteins in the complex. High resolution protein-protein docking was performed by the program PyDock [22]. PyDock is a method for rigid-body protein-protein docking. It explores either FTDOCK or ZDOCK methods to generate conformations of complexes and uses a scoring function that applies electrostatics and desolvation energies to select the best solutions.

The FPSPD program [42] was used for the identification of functional sites. FPSPD utilises environment-dependent substitution tables and evolutionary trace analysis to identify residues from a structurally-aligned homologous family of proteins that are unusually highly conserved. Solvent accessibility calculations are used to estimate the probability of residues and molecular patterns being directly involved in functional interactions. The three-dimensional structure is used to estimate the borders of the functional patterns.

### Plasmids and reagents

pcDNA3TLR4, pcDNA3CD14, pEFIREMD2, pNFBluc and pRantes were as previously described [43], [44]. Lipopolysaccharide (*Escherichia coli* serotype 0127;B8) was obtained from Sigma; recombinant human TNF and IFN were from R&D Systems. The phRGTK plasmid (Promega) was used as a transfection control. Site-directed mutagenesis was performed on pCDNA3TLR4 using the Stratagene Quikchange procedure.

### Cell culture, transfections and luciferase assay

HEK293 cells were maintained in Dulbecco's modified Eagle's medium supplemented with 10% fetal calf serum, 100 µg/ml penicillin/streptomycin and 2 mM L-glutamine at 37°C and 5% CO_2_. Cells were seeded at 10^5^ ml^−1^ 24 hr prior to transfection using Polyfect (Qiagen). Cells were stimulated 48 hrs post transfection. The Dual-Glo system from Promega was used for luciferase assays. Luminescence readings were obtained using a Lumister luminometer (BMG Labtech).

## Supporting Information

Table S1 Joy alignment key
(0.06 MB DOC)
