# Supplementary figures and images for: A Dimer of the Toll-Like Receptor 4 Cytoplasmic Domain Provides a Specific Scaffold for the Recruitment of Signalling Adaptor Proteins
Source: PLoS One. 2007 Aug 29;2(8):e788. doi: 10.1371/journal.pone.0000788 (PMC1945083; doi:10.1371/journal.pone.0000788)

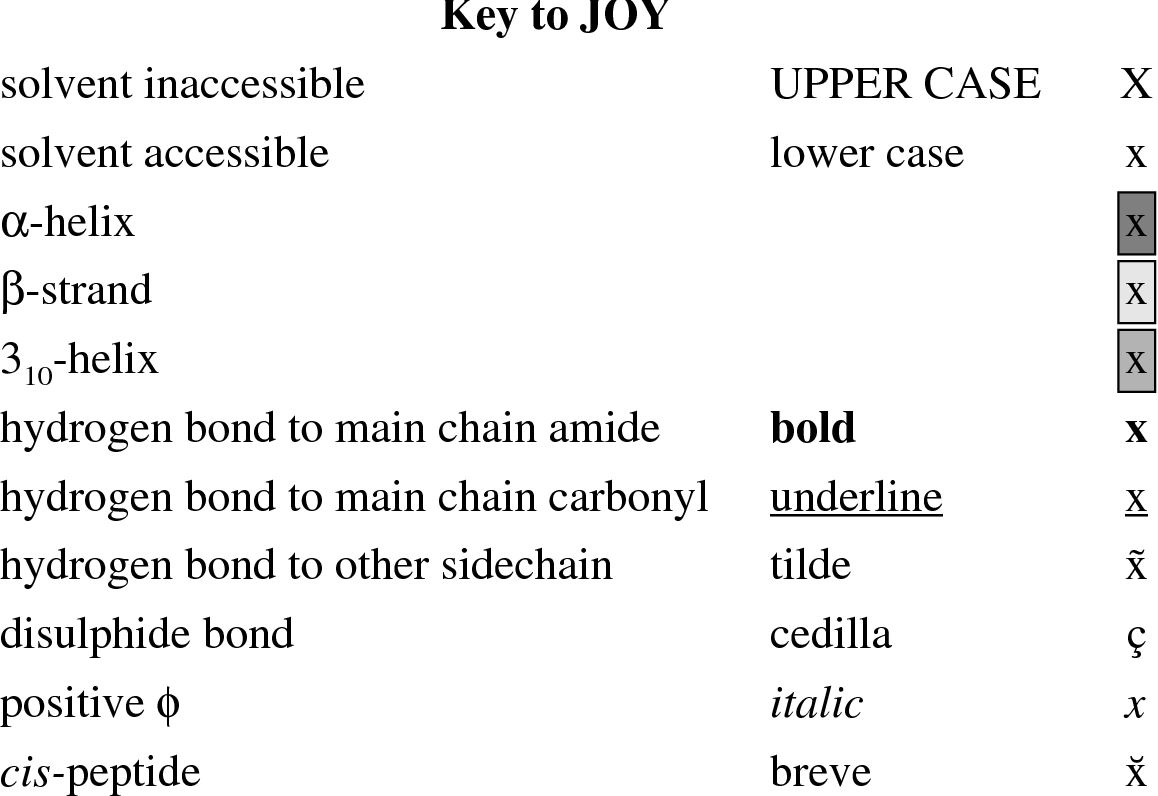

Supplement: Table S1 — Joy alignment key (0.06 MB DOC) [file pone.0000788.s001.doc]
